# Delivery of care in high mortality hospital settings: a direct observational study examining 1848 h of neonatal nursing in Kenya

**DOI:** 10.1016/j.eclinm.2025.103434

**Published:** 2025-08-14

**Authors:** Abdulazeez Imam, Michuki Maina, Jalemba Aluvaala, Vincent Kagonya, Onesmus Onyango, Fredrick Were, Sebastian Fuller, Kenneth Karumba, HIGH-Q Author Group, Peter Mwangi, Lucy Kinyua, Lydia Thuranira, Virginia Njoroge, Ngina Mwangi, Loise Mwangi, Penina Musyoka, Zainab Kioni, Mike English, David Gathara

**Affiliations:** a KEMRI-Wellcome Trust Research Programme, Nairobi, Kenya; b Health Systems Collaborative, Nuffield Department of Medicine, University of Oxford, S Parks Rd, Oxford, OX1 3SY, UK; c Department of Paediatrics and Child Health, University of Nairobi, Nairobi, Kenya; d Kenya Paediatric Research Consortium, Nairobi, Nairobi County, Kenya

**Keywords:** Small and sick newborns, Nursing, Quality of newborn care, Missed nursing care, Nursing workforce

## Abstract

**Background:**

In resource-constrained countries, deploying better technologies is expected to improve neonatal care but little attention has been paid to the critical role of nurse staffing. We investigate nursing workloads and their relationship with care provision in recently upgraded Kenyan neonatal units.

**Methods:**

We conducted a cross-sectional analysis using data from direct bedside observations across 8 intermediate-level neonatal units (defined by the World Health organization (WHO) as neonatal units capable of providing specialised but not intensive neonatal care) in Kenya over a 6-week period between February and March 2022. We excluded babies who were so severely ill that they were at risk of imminent death or transfer to a critical care facility and babies with congenital anomalies and surgical conditions. We used a structured observation checklist that had undergone content and face-validity testing in Kenya to collect workload and nursing care provision data. We determined nursing hours per patient per 12-h shift to measure nursing workload (primary exposure variable) and used a composite measure, the nursing care index (NCI), to score the nursing care delivered to a baby (primary outcome variable). The relationship between nursing workload and nursing care provision was assessed using multilevel models.

**Findings:**

Across 8 hospitals spanning 1848 h of observation of 597 sick newborns, the median nursing time available for each newborn ranged from 19.2 to 72.0 min on a 12-h shift. Nurses delivered 32% of expected care, completely missed 32%, and informally delegated 36% of tasks. Unsupervised nursing students and mothers played prominent roles in the care of clinically unstable babies. An exploratory model combining qualified nurse and nursing student hours, that considerably expanded the range of the nursing workload metric, demonstrated a 3.1% increase in nurse-delivered care per additional 60 min of nursing time per baby per shift (β: 0.031, 95% CI: 0.019–0.043).

**Interpretation:**

The nursing time available to care for sick newborns in Kenya is inadequate resulting in suboptimal care quality. Improving care quality, reducing of newborn mortality and making effective use of new technologies may not be possible without increasing nurse staffing in resource-constrained countries.

**Funding:**

10.13039/501100000272National Institute for Health and Care Research.

**Research in context**

**Evidence before this study**

The most significant nursing workforce shortages are in resource-constrained low-middle income countries (LMICs), with limited data available to demonstrate the effect of inadequate staffing or heavy workloads on the quality of patient care. While increasing access to technology and providing adequate human resources for health are both crucial for improving neonatal care quality and reducing neonatal mortality, there has been insufficient focus on the latter in low-resource neonatal care settings.

Before conducting this study, we conducted and published two quantitative systematic literature reviews (Imam et al., 2022 and Imam et al., 2023). We found one published paper by Gathara et al. that reported on staff-to-baby ratios and their association with quality of care in a neonatal care setting. This paper was mainly focused on particular care that was missed and not on the nuanced details of care provision within neonatal units in high-workload LMIC contexts.

**Added value of this study**

We provide the largest published study of observed nursing care provision in LMIC hospitals, showing high levels of missed professional nursing care and ‘off-loading’ of care to nursing students and mothers who were poorly prepared for and unsupervised when providing care because of severe nursing shortages. We also show a clear relationship between the time available to provide care for each baby and the care delivered was apparent in these high-workload settings.

**Implications of all the available evidence**

Improvements in neonatal nurse staffing and nursing care delivery are lagging the current drive to leverage technology and other improvement strategies to extend access to intermediate-level neonatal care and reduce neonatal mortality globally. Nurse staffing will need to be substantially improved to maximise the gains of improving access to neonatal care.

Our findings provide important evidence in support of the post-2025 Every Newborn Action Plan by United Nations Children's Fund and WHO that proposes specific standards for neonatal nurse-to-patient ratios but also demonstrates the scale of the challenge faced in meeting these standards.

## Introduction

It is estimated that at least three million lives might be saved annually by improving the quality of care in the newborn and immediate postnatal period, particularly in low and middle-income countries (LMICs).^1^ To improve small and sick newborns (SSNC) survival, target 3.2 of the Sustainable Development Goals (SDGs) aims to reduce the global neonatal mortality rate to under 12 per 1000 live births.^2^ To achieve this target, the World Health Organization (WHO) in 2014 launched the Every Newborn Action Plan (ENAP), now implemented by 106 countries, as a roadmap for ending preventable newborn deaths through investing in peripartum care, improving quality and access to maternal and newborn care, leveraging the power of families and community and tracking birth outcomes and care quality around birth.^3^ Recognising the slow pace in attaining the neonatal SDG target, there has been a recent focus on increasing intermediate-level neonatal unit (defined by WHO as neonatal units capable of providing specialised but not intensive neonatal care) access with a target that by 2025, 80% of districts (at the sub-national level) in every country should have at least one of such units.^4^

Historically, however, access to care has not always meant quality care delivery, particularly in LMICs.^5^ A key limiting factor is human resources for health; recognised as central to improving health facility quality of care by key global policy documents.^6^^,^^7^ Post-2025, additional ENAP targets are being planned including minimum neonatal nursing numbers for intermediate-level neonatal units, recognising their key roles in ensuring better SSNC outcomes.

Increasing neonatal unit nurse staffing is being proposed in the face of major nursing workforce deficits and consequently high nursing workloads in many LMICs.^8^ In these settings, high nursing workloads are normalised without evidence of their effects on patient care quality and may continue to be tolerated despite ENAP recommendations.^9^ We aimed to address this knowledge gap, employing direct bedside observations of care in Kenyan neonatal units being upgraded to intermediate-level capacity through the provision of essential technologies, training and mentorship,^10^ we examine nursing workloads in detail and investigate their relationships with nursing care provision (an indicator of quality care).

## Methods

### Study design

This was a cross-sectional analysis using data derived from direct bedside observations of nursing care provided to newborns admitted in intermediate-level neonatal units in Kenya over a six-week period between February and March 2022. This study is part of a wider body of work known as the Harnessing Innovations in Global Health for Quality Care (HIGH-Q) programme.^11^

### Ethical approval and consent to participate

Ethical approval for this study has been obtained from the Kenyan Medical Research Institute (KEMRI) Scientific and Ethics Review Unit (KEMRI/RES/7/3/1) and the University of Oxford Research Ethics Committee (26–21). Written informed consent for this study was obtained from the caregivers of babies who were observed and from nurses carrying out care for these babies.

### Research setting

The study was conducted in eight Kenyan intermediate newborn care units with overall mortality rates of approximately 10%.^12^ These provide respiratory support with continuous positive airway pressure (CPAP) but not more advanced modes of ventilation and care for SSNC.^13^ They were all government-owned units linked to busy maternity wards providing Comprehensive Emergency Obstetric and Neonatal Care and serve as nursing students' clinical training units.

### Study population

To capture all shift types, we used stratified random sampling to identify 12-h day and night shifts on weekdays and weekends. We used existing Kenyan guidance to allocate sampled shifts in advance focusing on 3 illness severity categories aiming to observe a balanced number of newborns across categories spanning: Category A (unstable) babies requiring support with vital functions, for example, babies on CPAP; Category B (stable but ill) babies on interventions such as intravenous medication and nasogastric tube feeding; and Category C, babies who are otherwise stable but transitioned to oral antibiotics or on the unit for kangaroo mother care.1. As babies in these categories are typically co-located in Kenyan newborn units, one individual could observe care delivered for up to four babies of the same care category per shift. Observation blocks were thus stratified into day or night in a 50:50 ratio, and weekends and weekdays in a 2:4 ratio and a sequence of random nursing shift blocks was created for each baby category.

We excluded babies who were so severely ill that they were at risk of imminent death or transfer to a critical care facility and babies with congenital anomalies and surgical conditions. The latter group have different observation and treatment standards from those defined in Kenyan nursing guidelines.

### Variables

#### Data sources/measurements

We used a structured observation checklist that had undergone content and face validity testing in Kenya to collect workload and nursing care provision data.^14^ Using this tool, we collected data on shift-level staffing and patient-facing nursing care provided to babies (Supplementary File S1).

### Data collection processes

We conducted pilot studies to determine the feasibility of our data collection processes. We trained eight nutritionists as data collectors (observers), intending that they would understand the hospital context and medical jargon, be less influenced by professional loyalties, and be better at retaining non-participant status during observations.

We developed standard operating procedures for data collection, trained the observers as one group in a non-study hospital, and began study site-specific observations with one week of supervised pilot-testing of the data collection processes in their respective neonatal units. During this week, to promote reliability, observations made by trained observers and those independently conducted by senior research team members (who included nurses) were compared, and correct responses were clarified in line with written standard operating procedures. Our one-week supervised piloting also allowed the observers to familiarise themselves with unit layouts and ways of working and permitted nurses to become comfortable with being observed, potentially minimising the Hawthorne effect.^15^ We further minimised the Hawthorne effect by spreading data collection over six weeks and across randomly selected 12-h nursing shifts.

Each observer observed nursing care for 3 to 4 co-located babies of the same illness severity (category A, B or C) using our structured observation tool. In this way, the observers could check whether the set number of expected tasks they had been trained to identify were performed for the babies they observed while identifying who performed these tasks. This was found to be logistically feasible in our pilot study and our earlier published research.^14^

### Study size considerations

We calculated sample size for proportions, setting our confidence level at 95%, and precision at 5% and determined the proportion of nurse-delivered care (our primary outcome) to be 42.4% based on a previously published Kenyan study.^14^ Assuming a design effect of 1.5 to allow for the clustering of babies observed on a shift, we determined a minimum sample size of 563 babies.

### Quantitative variables

#### Exposure variables

Our primary exposure variable is a measure of nursing workload, the nursing hours per patient per 12-h shift.^16^ This metric sums up all nursing care hours available on a 12-h nursing shift (for example 2 nurses on a 12 h shift will contribute 24 nursing hours), and then divides this by the number of babies present at the shift start, creating an average time available per baby (as opposed to a nurse-to-patient ratio).^16^ Our measure was calculated assuming nurses take no breaks. Clearly, nurses’ work includes much more than direct bedside care, including many ward management roles amongst others, and so the average time available per baby for direct bedside patient care would be lower. To calculate this metric, we combined nursing hours on all individual shift types during the day (morning, afternoon or day (Table 1), while night shifts were single shifts (Table 1). We standardised the metric (both night and day) to a 12-h shift pattern to allow for uniform comparison across neonatal units. Shifts with heavy or low workloads would have less or more average nursing time available per baby, respectively. Given the small number of nurses per shift, we derived a second exposure variable that included nurses' and nursing students’ hours to calculate the combined nursing hours per patient per 12-h shift. This allowed for investigating associations with nursing care provision over a larger range of nursing hours.

**Table 1:** Bed capacity, nurse staffing, patient number, equipment numbers and shift patterns compared across the 8 study neonatal units.

Domain	Variable	H1	H2	H3	H4	H5	H6	H7	H8
Physical structure	Bed capacity^b^	50	53	47	38	50	71	28	26
Number of babies	Median (IQR) number of admitted babies on a shift	60 (56–64)	42 (36–50)	23 (20–25)	23 (21–24)	53 (51–57)	55 (51.5–57)	20 (20–24)	38 (34–39)
Nurse staff strength	Nurse numbers (total headcount)	11	17	8	10	14	13	10	12
	Average shift level Nurse-to-patient ratios^c^	1:29	1:21	1:22	1:16	1:27	1:26	1:10	1:38
	Ward assistants^a^	3	3	1	1	6	3	2	1
Nursing students median (IQR)	Number of nursing students on a shift	2 (2–5)	6 (4–10)	6 (3–10)	8 (4–12)	7 (5–21)	0.5 (0–2)	3 (2–4)	8 (5–16)
Nurse skill mix (%)	Masters	0 (0.0)	0 (0.0)	0 (0.0)	0 (0.0)	0 (0.0)	1 (7.7)	0 (0.0)	0 (0.0)
	Bachelors	1 (9.1)	2 (11.8)	0 (0.0)	0 (0.0)	1 (7.1)	1 (7.7)	1 (10.0)	0 (0.0)
	Higher Diploma	2 (18.2)	6 (35.3)	0 (0.0)	1 (10.0)	3 (21.4)	2 (15.4)	2 (20.0)	2 (16.7)
	Diploma	7 (63.6)	7 (41.2)	7 (87.5)	9 (90.0)	11 (78.6)	8 (61.5)	7 (70.0)	10 (83.3)
	certificate	1 (9.1)	2 (11.8)	1 (12.5)	0 (0.0)	0 (0.0)	1 (7.7)	0 (0.0)	0 (0.0)
Number of functional equipment for essential newborn care and neonatal resuscitation	Radiant warmers	3	3	3	3	9	4	1	3
	Suction machines	3	4	3	3	2	2	2	2
	Glucometers	2	1	0	1	3	1	1	1
	Incubators	8	7	3	5	12	11	4	2
	CPAP machines	9	3	2	3	7	6	2	2
	Phototherapy	5	5	2	5	7	6	6	5
Shift pattern and length (hours)	Morning shift	NA	5	NA	5	6	NA	NA	5
	Afternoon shift^d^	NA	6	6	6	5	6	5.5	6
	Day shift^d^	10	NA	11	NA	NA	9	9	NA
	Night shift	14	13	13	13	13	13	13	13

CPAP is Continuous Positive Airway Pressure, IQR is interquartile range, H1–H8 represent each of the 8 neonatal units.

a Ward assistants had non-patient facing tasks, including tasks such as cleaning the neonatal unit environment, collecting unit supplies. NA means Not applicable.

b In some of the neonatal units, the unit occupancy is >100% as up to 2–3 babies sharing a bed/incubator.

c This presents average ratios across day, night, weekday and weekend nursing shifts and is derived from the average nursing hours per patient per 12 h shift. Morning shift is from 07:30 to 12:30 except in H5 where it is 07:30 to 13:30. Afternoon shift is 12:30 to 18:30 except in H5 and H7 where it is 13:30 to 18:30 and 12:30 to 18:00 respectively. Day shift is 07:30 to 16:30 except in H1 and H3 where it is 07:30 to 17:30 and 07:30 to 18:30, respectively.

d Day and afternoon shifts overlap for H3, H6 and H7.

#### Outcome variable

As a metric of care delivered, we used a composite measure, the nursing care index (NCI), an unweighted patient-level aggregate score of nursing care delivered to a baby based on the observational checklist (Supplementary File S1). It is expressed as a proportion calculated from the numerator of observed care divided by all expected care that should be provided to a baby.^14^ Expected nursing care is based on a baby’s individual medical and nursing needs derived from the interventions each baby is receiving and the minimum standards for newborn nursing care in Kenya.^17^ Expected care varies with patient illness severity; for example, Category A babies require greater frequency of care related to receipt of more specific interventions (e.g., oxygen and intravenous fluids) and more frequent vital signs monitoring. When observations were truncated, for example, when a baby was discharged, the index's denominator (expected care) was calculated based on the care expected during the actual hours observed.

#### Deriving the outcome (Nursing care index) variable

We used previously published methods to derive the individual NCI for a baby.^14^ To do this, responses from the structured observational checklist were scored as 1 (when a task was carried out) and 0 (when not conducted). We summed up scores across all observed nursing tasks to derive a total for each baby which was then divided by the total expected score to derive the proportion of nursing care conducted (NCI). We created two outcome scores:

a. An NCI based on solely nurse-delivered care (nNCI) representing tasks performed by a qualified nurse or a nursing student directly supervised by a nurse. Any tasks done by an unsupervised student or any other person were classed as not done using this indicator.
b. An NCI based on tasks nurses, supervised students and unsupervised nursing students performed (sNCI) representing tasks performed by either of these categories but classifying any tasks done by any other person as not done.

In both cases, the denominator was expected nursing care calculated at the individual baby level. Observed scores are therefore smaller for the nNCI than the sNCI.

### Statistical methods

#### Data analysis

We use frequencies and percentages to provide summary statistics for individual nursing tasks and summary statistics and graphical displays (box plots) to summarise average nursing workloads measured using our primary and secondary nursing workload (exposure) variables and our outcome variables (nNCI and sNCI). We examined the relationship between exposure and outcome variables using scatterplots. We handled missing data using complete case analysis.

Recognising the hierarchical nature of our data (observed babies nested in shifts which were nested within neonatal units), we employed multilevel modelling to investigate how changes in nursing workload affected nursing care provision. We investigated two models–the effect of nursing hours per patient per 12-h shift on nurse-delivered care (nNCI) and that of the combined nursing and student hours per patient per 12-h shift on student and nurse-delivered care (sNCI). We used Akaike’s Information Criteria (AIC), Bayesian Information Criteria (BIC) and Likelihood ratio tests (LRT), to determine the best-fit models for our analysis. We built our models by determining the best base model using the AIC, BIC, and LRT and sequentially added covariates until we determined the best-fit model. We compared fit statistics for a linear regression model, an unadjusted two-level random intercept model and a two-level random intercept model adjusted for confounders. We also checked the model assumptions for normality and homoskedasticity. All our data analysis was conducted in STATA version 18. Our model equation is specified below:

Yij = β_o_ + β_1_ x_ij_ + μ_j_ + e_ij_, where x_ij_ represents the ‘ith’ baby in the ‘jth’ neonatal unit. The dependent variable Y represents nurse-delivered care, while X represents the combined nursing hours per patient per 12 h. μ represents the cluster level (hospital) residuals, while e represents the residuals at the individual level.

### Patient and public involvement

The HIGH-Q programme involved County Directorates of Health and Hospital Management Teams in the study implementation. Before the study design, the project involved the Nursing Council of Kenya (NCK) and the National Nurses Association of Kenya (NNAK) in discussing the challenge of nursing workforce shortages and the need to properly document the impact of this for policy advocacy. At multiple stages of the project, the preliminary findings and final study results were fed back to stakeholders involving local hospital teams, hospital management teams, nursing professional bodies, development partners and civil society organisations.

### Role of funding sources

The study sponsors had no role in study design, data collection, analysis, interpretation of data, the writing of the report; and in the decision to submit the paper for publication.

## Results

Our study units were of varying sizes (ranging between 26 and 71 beds) and were poorly staffed. For example, one hospital (H3) assigned eight nurses to provide 24-h, 7-day-a-week care for a 47-bed neonatal unit. Even the best-staffed hospital (H2) had 17 nurses for its 53-bed unit (Table 1). Most nurses were trained at diploma level and not specialised nurses and were supported by small numbers of ward assistants who had non-patient-facing roles (Table 1). Larger units had more equipment and technologies (e.g., CPAP) but deficits in many resources to support basic newborn care (for example, glucometers).

**Fig. 1:**
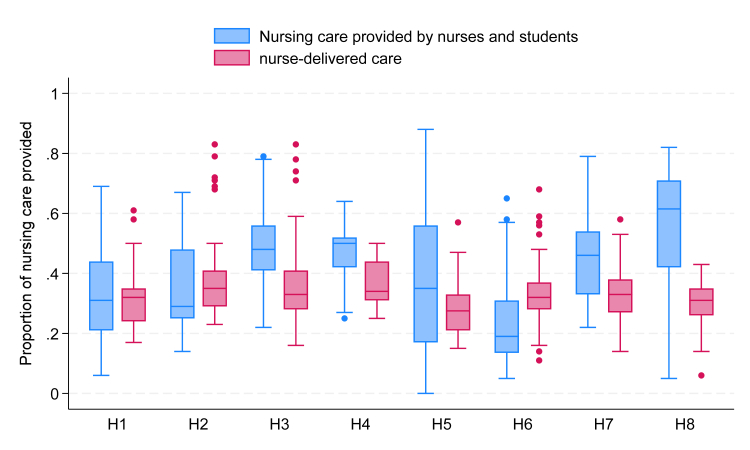
Box plots showing nurse-delivered (nNCI) and nursing care provided by nurses and students (sNCI) to babies across neonatal units on observed nursing shifts (H1–H8) (n = 597). The boxes represent the middle 50% of the data, while the whiskers represent the top and bottom 25%.

We observed bedside nursing care for 597 babies across 154 12-h nursing shifts, making 1848 h of observation. On average, across all the neonatal units combined, nurses delivered 32% (Interquartile range (IQR)—27–38)) of the nursing care we measured, 36% of care was performed by others (mainly nursing students and mothers), and the rest (32%) of care was completely missed. More severely ill babies (Category A) have more expected tasks than less severely ill babies (Category C). Nurses on average provided 33% (IQR 26–40) of expected care for category A babies, 29% (IQR 26–33) for category B babies and 35% (IQR- 29–40) for category C babies. Nurse-delivered care (nNCI) was similar across the eight neonatal units, while the combined nurse and nursing student-delivered care (sNCI) varied more substantially, with the greatest student involvement in nursing care seen in H8 (Fig. 1).

**Fig. 2:**
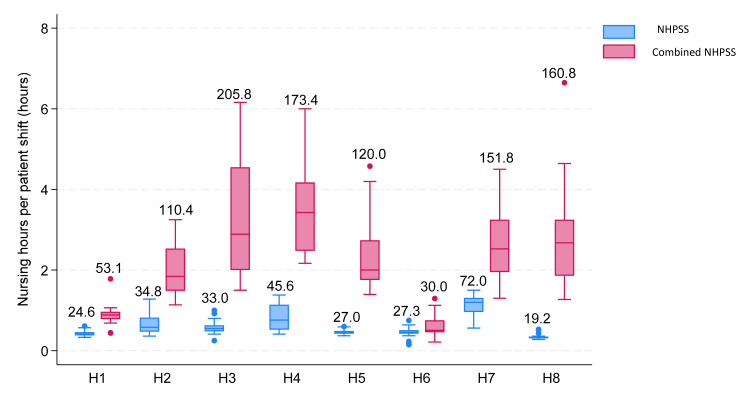
Shift-level nursing input measured as nursing hours per patient per 12-h shift across eight neonatal units (H1–H8). (n = 154). NHPSS—Nursing hours per patient per 12-h shift, Combined NHPSS–Combined nursing hours per patient per 12-h shift. The boxes represent the middle 50% of the data, while the whiskers represent the top and bottom 25%. The legends above each boxplot represent the median nursing hours translated to minutes.

The median nursing hours per patient per 12-h shift (our primary nursing workload measure), pooled across all shifts and sites, was 0.51 h per patient; equivalent to 30 min (Interquartile range (IQR)—0.41–0.68 h). This ranged from 0.32 nursing hours per patient in H8 (19.2 min) to 1.20 nursing hours per patient in H7 (72.0 min) (Fig. 2). It also varied across day (0.51 (30 min), IQR—0.43–0.68) and night shifts (0.46 (27.6 min), IQR—0.39–0.68), as well as weekday (0.51 (30 min), IQR—0.43–0.63) and weekend shifts (0.46 (27.6 min), IQR—0.37–0.78). The nursing metric correlated poorly with nurse-delivered care (nNCI, r = 0.14). Our secondary nursing workload measure, the combined nursing hours per 12-h shift (including student time), varied more substantially across the neonatal units, ranging from a median of 0.50 (30 min), IQR—0.46–0.75 in H6 to 3.43 (205.8 min), IQR—2.48–4.17 in H3, and correlated strongly with the secondary outcome of nurse and nursing student-delivered care (sNCI, r = 0.48, Supplementary File S2).

Analysing specific task completion indicates nurses prioritised some items of care for the sickest babies (category A) over more stable babies (category B and C). For example, while they clinically assessed 90.1% (182/202) of category A babies before the start of a shift, they did so for 83.3% (155/187) and 70.2% (146/208) of category B and C babies, respectively (Table 2). They also measured the first temperatures for 22.5% (45/200), 3.2% (6/186), and 2.4% (5/208) of category A, B, and C, respectively (Table 2). This pattern of prioritisation was observed across multiple domains of nursing care (Table 2). Conversely, nurses deprioritised basic nursing care that was commonly observed to be delegated to unsupervised mothers. For example, tasks such as cleaning babies, cord care, and feeding, including nasogastric tube feeding, were performed on fewer than 5% of occasions by nurses (Table 2 and Supplementary File S3). Nurses also delegated critical roles to nursing students with no direct supervision, for example, students administered 50.7% (34/67) of intravenous medications for the sickest babies (category A) and conducted approximately half of all vital sign monitoring (Table 2).

**Table 2:** Specific nursing tasks disaggregated by patient care category and whether the task was performed by the nurse, nursing student, or mother or was missed^a^ (n = 597).

Domains	Task	Category A babies				Category B babies				Category C babies			
		n	Nurse	Others (Nursing student/mother)	Missed	n	Nurse	Others (Nursing student/mother)	Missed	n	Nurse	Others (Nursing student/mother)	Missed
Routine nursing care	Patient assessment before a shift	202	**90.1** [57.3, 98.4]	0.0 [0.0, 0.0]	9.9 [1.6, 42.7]	186	**83.3** [44.8, 96.9]	0.0 [0.0, 0.0]	16.7 [3.1, 55.2]	208	**70.2** [42.0, 88.5]	0.0 [0.0, 0.0]	29.8 [11.5, 58.0]
	Ward round attendance	68	**22.1** [10.2, 41.4]	0.0 [0.0, 0.0]	77.9 [58.6, 89.8]	44	**11.4** [1.3, 56.2]	0.0 [0.0, 0.0]	88.6 [43.8, 98.7]	44	**6.8** [1.1, 33.1]	0.0 [0.0, 0.0]	93.2 [66.9, 98.9]
Vital sign monitoring^a^	Temperature	200	**22.5** [7.8, 50.0]	50.5 [26.9, 73.9]	27.0 [13.0, 47.9]	186	**3.2** [0.9, 11.3]	60.8 [28.1, 86.0]	36.0 [12.2, 69.5]	208	**2.4** [0.3, 14.9]	52.9 [26.7, 77.6]	44.7 [19.9, 72.4]
	Pulse oximetry	177	**33.3** [15.3, 58.1]	48.0 [23.1, 74.0]	18.6 [8.3, 36.7]	171	**4.1** [1.4, 11.3]	57.9 [23.1, 86.3]	38.0 [11.0, 75.3]	184	**4.3** [1.1, 16.2]	41.8 [13.7, 76.6]	53.8 [20.3, 84.2]
Routine newborn care^b^	Cleaning baby	152	**0.7** [0.1, 6.7]	67.1 [41.1, 85.6]	32.2 [13.6, 59.0]	132	**0.0** [0.0, 0.0]	60.6 [24.9, 87.7]	39.4 [12.3, 75.1]	137	**0.7** [0.1, 7.2]	59.1 [23.8, 87.0]	40.1 [12.6, 75.8]
	Cord care	138	**0.7** [0.1, 8.7]	53.6 [29.3, 76.4]	45.7 [22.8, 70.6]	115	**1.7** [0.4, 8.0]	53.9 [26.2, 79.4]	44.3 [18.9, 73.2]	91	**0.0** [0.0, 0.0]	64.8 [24.4, 91.3]	35.2 [8.7, 75.6]
Physical turning^a^	Physical turning	200	**3.5** [1.1, 10.3]	90.5 [75.0, 96.8]	6.0 [1.5, 20.9]	186	**1.6** [0.3, 8.2]	97.3 [91.3, 99.2]	1.1 [0.2, 4.7]	207	**1.4** [0.5, 4.4]	97.6 [90.8, 99.4]	1.0 [0.1, 9.3]
Cup feeding^a^	Cup feeding	15	**6.7** [0.8, 39.7]	93.3 [60.3, 99.2]	0.0 [0.0, 0.0]	64	**1.6** [0.2, 10.6]	96.9 [88.1, 99.3]	1.6 [0.2, 10.6]	98	**4.1** [1.5, 10.5]	93.9 [86.9, 97.3]	2.0 [0.5, 7.9]
Nasogastric tube feeding^a^	Nasogastric tube feeding	141	**0.0** [0.0, 0.0]	95.0 [89.9, 97.6]	5.0 [2.4, 10.1]	53	**0.0** [0.0, 0.0]	98.1 [87.3, 99.7]	1.9 [0.3, 12.7]	0	**0.0** [0.0, 0.0]	0.0 [0.0, 0.0]	0.0 [0.0, 0.0]
Medication	Oral medication^a^	31	**9.7** [3.0, 27.0]	61.3 [42.7, 77.1]	29.0 [15.4, 47.9]	12	**16.7** [3.5, 52.4]	75.0 [40.9, 92.9]	8.3 [0.9, 47.5]	31	**6.5** [0.2, 23.5]	67.7 [48.9, 82.2]	25.8 [13.0, 44.6]
	Intravenous medication^b^	67	**40.3** [29.1, 52.6]	50.7 [38.7, 62.7]	9.0 [4.0, 18.8]	74	**54.1** [42.5, 65.2]	36.5 [26.2, 48.2]	9.5 [4.5, 18.7]	0	**0.0** [0.0, 0.0]	0.0 [0.0, 0.0]	0.0 [0.0, 0.0]
Continuous Positive Airway Pressure^a^	Checking nasal prong position	13	**38.5** [15.3, 68.4]	46.2 [20.3, 74.2]	15.4 [3.3, 49.3]	0	**0.0** [0.0, 0.0]	0.0 [0.0, 0.0]	0.0 [0.0, 0.0]	0	**0.0** [0.0, 0.0]	0.0 [0.0, 0.0]	0.0 [0.0, 0.0]
	Checking oxygen flow rate	13	**53.8** [25.8, 79.7]	15.4 [3.3, 49.3]	30.8 [10.7, 62.2]	0	**0.0** [0.0, 0.0]	0.0 [0.0, 0.0]	0.0 [0.0, 0.0]	0	**0.0** [0.0, 0.0]	0.0 [0.0, 0.0]	0.0 [0.0, 0.0]
Oxygen therapy^a^	Checking nasal prong position	64	**20.3** [12.0, 32.2]	78.1 [66.1, 86.7]	1.6 [0.2, 10.6]	0	**0.0** [0.0, 0.0]	0.0 [0.0, 0.0]	0.0 [0.0, 0.0]	0	**0.0** [0.0, 0.0]	0.0 [0.0, 0.0]	0.0 [0.0, 0.0]
	Checking oxygen flow rate	65	**21.5** [13.1, 33.4]	55.4 [43.0, 67.1]	23.1 [14.3, 35.1]	0	**0.0** [0.0, 0.0]	0.0 [0.0, 0.0]	0.0 [0.0, 0.0]	0	**0.0** [0.0, 0.0]	0.0 [0.0, 0.0]	0.0 [0.0, 0.0]

NA—Not applicable. When ‘a’ is written next to the variable, this represents the other group is mothers, when b is written, the other group are nursing students.

Emboldened numbers in the table show the percentage of tasks performed by nurses for each category of baby and allow for visual comparison across categories.

Numbers in parentheses within rows contain cluster (hospital) adjusted 95% confidence intervals.

a Selection of individual tasks made to illustrate varied patterns, full table available in Supplementary File S2.

Using Multilevel models, we found no significant association between nursing hours per patient per 12-h shift (our primary exposure metric) and nurse-delivered care (adjusted coefficient −0.020 (95% CI −0.059, 0.019), Table 3)). The combined nurse and nurse student hours per patient per 12-h shift (our secondary exposure metric) was associated with a 3.1% increase in care provision by nurses and nursing students (coefficient 0.031 (0.019, 0.043) for every hour increase in this staffing metric (Table 3). Our model met assumptions for normality and homoskedasticity (Supplementary Files S4 and S5). The intraclass correlation coefficients (95% confidence interval) for our primary outcome model (Model 1) and our secondary outcome model (Model 2) were 0.120 (0.039, 0.313) and 0.184 (0.066, 0.418), respectively (Table 3).

**Table 3:** Multilevel models (Gaussian) examining the relationship between nursing workload and nursing care delivery (n = 597).

Characteristics	Nurse-delivered care			Nurse and nurse student delivered care		
	Model 1	Model 2	Model 3^a^	Model 1	Model 2	Model 3^a^
	B-coefficient (95% confidence interval)	B-coefficient (95% confidence interval)	B-coefficient (95% confidence interval)	B-coefficient (95% confidence interval)	B-coefficient (95% confidence interval)	B-coefficient (95% confidence interval)
Nursing hours per patient per 12-h shift^b^	0.081 (0.035, 0.128)	−0.004 (−0.043, 0.035)	−0.020 (−0.059, 0.019)	0.060 (0.050, 0.070)	0.035 (0.022, 0.049)	0.031 (0.019, 0.043)
Patient illness severity						
Category A			Reference			Reference
Category B			−0.056 (−0.076, −0.037)			−0.063 (−0.091, −0.034)
Category C			−0.014 (−0.033, 0.005)			−0.167 (−0.194, −0.139)
Model fit statistics						
AIC	−386.60	−1009.77	−1023.84	−446.28	−469.01	−580.45
BIC	−377.82	−992.21	−997.49	−437.49	−451.44	−554.10
LRT (P-value comparing model to a linear model)		<0.001	<0.001		<0.001	<0.001
Variance						
Hospital-level		0.0012 (0.0004, 0.0041)	0.0014 (0.0004, 0.0045)		0.0043 (0.0013, 0.0140)	0.0045 (0.0014, 0.0143)
Baby-level		0.0101 (0.0090, 0.0114)	0.0096 (0.0086, 0.0108)		0.0250 (0.0222, 0.0279)	0.0201 (0.0179, 0.0225)
Intraclass correlation coefficient		0.109 (0.035, 0.291)	0.120 (0.039, 0.313)		0.147 (0.050, 0.361)	0.184 (0.066, 0.418)

Model 1 is a linear regression model, Model 2 is an unadjusted multilevel regression model based on a two-level random intercept model (Level 1—Baby and level 2 is hospital), Model 3 is the two-level model adjusted for patient illness severity.

LRT—Likelihood ratio test, AIC—Akaike’s Information Criteria, BIC—Bayesian Information Criteria.

a Best fit model for each outcome.

b Nursing hours per patient per 12 h shift is combined nurse and nursing student hours per 12 h shift for the Nurse and nurse student delivered care outcome.

## Discussion

While it is recognised that nursing workloads are high in low-resource newborn settings, the impact of this on care provision and quality of newborn care remains poorly studied. Our nursing workload metric, the median nursing hours per patient per 12-h shift (NHPPS), which determined the average time available per baby on a nursing shift (assuming no breaks are taken) across our eight study units, was 0.51 h (IQR—0.41–0.68), equivalent to 30.6 min per patient. This ranged between 19.2 and 72 min per baby across our eight study neonatal units. At this level of nursing workload, on average, nurses managed to provide 32% of the expected bedside nursing care for 597 babies across eight neonatal units. They delegated 36% of nursing care to nursing students and mothers and 32% care was completely missed. This level of nursing workload starkly contrasts with an earlier US study, which reported an average NHPPS in intermediate-level units of 5.43 h (325.8 min)^16^; a 10-fold difference. Although in practice, nurses are likely to spend varying amounts of time with babies depending on individual needs, the NHPPS provides an average based on total nursing time available and the number of babies in neonatal units. We believe it helps focus attention on what are urgent staffing concerns that have remained unaddressed for many years. While Kenya has guidance on neonatal unit nurse staffing linked to unit size, it has no regulated policy.^18^ This might explain some of the variation seen within newborn units in this context and other low-resource settings.^19^ In high-income settings, neonatal nurse staffing is guided by policies promoting patient safety. In the UK, for example, ratios of 1:2 and 1:4 nurses per baby are recommended for high dependency and special care, respectively, the forms of care provided by intermediate-level Kenyan units.^20^

Our quantitative data demonstrate how nurse-provided bedside care is influenced in high-workload, low-resource neonatal units, complementing prior qualitative studies.^21^^,^^22^ Nurses provided 33%, 29% and 35% of expected care for category A, B and C babies, respectively. This finding should be interpreted in the context of differences in our expected nursing care denominator for patients with varying illness categories. The sickest babies (category A) require significantly more nursing tasks when compared to stable babies (Category C). For example, the sickest babies require more vital sign monitoring and would receive more interventions such as CPAP, intravenous fluids and oxygen. Disaggregating the data and examining its components across the three illness severity categories, we observed nurses prioritising technical aspects of care for the sickest of babies. For example, nurses measured first temperatures on 22.5% of critically ill babies compared to just 2.4% for stable babies. Less technical tasks, such as cord care and feeding, which provide opportunities for engagement with and education of mothers, were deprioritised by nurses. Our data thus show how holistic nursing care is hugely undermined in these settings, with nurses triaging their care due to competing demands for their limited time.^22^^,^^23^ Some forms of care, for example, nasogastric tube feeding, a complex task with important safety concerns, were not prioritised by nurses and were mostly performed by unsupervised mothers, even for the sickest babies.^24^ These mothers often missed important steps of nasogastric tube feeding, including checking to see if the tube was correctly placed before feeding.

This highlights important quality and safety issues as nurses with very limited time do not formally delegate such tasks after training and confirming competency of mothers, rather these tasks are typically offloaded with minimal supervision. Similarly, nursing students who often spend only 2–4 weeks in neonatal units during their entire training undertook many tasks without direct supervision, including caring for critically ill babies on CPAP and oxygen and giving intravenous medications. Reliance on unsupervised nursing students was apparent in our data (with unpublished observations indicating supernumerary nurse tutors were very rarely present on neonatal units). On average, the more nursing students a unit had, the greater the proportion of nursing care that was delivered by either a qualified nurse or a student.

The task-shifting practices described here are not formalised or evidence-based and appear normative, with the same patterns observed across sites, likely arising from systemic maladaptation to chronic nurse staffing shortages perpetuated by the socialisation of student nurses in such contexts during their training. As noted by our observers in post-research debriefing exercises and supported by complementary ethnographic work,^25^ it seemed mothers and students based their practice more on peer-to-peer learning than expert demonstration and support, as has been reported elsewhere.^26^^,^^27^ This deviates from the ideal of family-centred care, which supports and works collaboratively with families in voluntary, shared care provision.^28^ The recent WHO/UNICEF comprehensive model for scale-up of small and sick newborn care (SSNC) at the district level (intermediate level) highlights the crucial role that family involvement and support should play as part of a core global strategy for improving neonatal survival.^29^ Our research highlights how nursing workforce deficits will undermine implementation of such recommendations, with nurses unable to support mothers but instead having to rely on them as an informal part of the workforce in neonatal units.

We found no association between nursing hours per patient per 12-h shift and nurse-delivered care (nNCI, relative risk: −0.020 (95%CI: −0.059, 0.019)). This nursing workload metric was extremely low and showed minimal variation across our eight neonatal units. Sites also had similar levels of nurse-delivered care with a consistent pattern suggesting a strong, shared normative influence that might help explain this result. When we examined the combined nurse and nursing student hours as an explanatory factor, which showed a 4-fold greater variation in this 12-h shift metric, there was a 3.1% increase in care delivery with every hour increase in nurse plus student nurse time. One interpretation of these findings is that we are unlikely to see any improvement in high-quality care for sick newborns delivered by professional nurses until staffing levels are higher than those seen in our best-staffed site, with at least 1.2 h per baby per 12-h shift. Data from the combined nursing and nurse student metric suggests staffing will need to be considerably higher than this to enable better delivery of skilled bedside nursing care. Such findings are consistent with earlier work in Kenyan private-sector neonatal units, where much better nurse staffing achieved higher levels of nurse-delivered care and are consistent with the normative guidance in high-income settings.^14^

Our findings have important implications. LMICs and their partners are making considerable efforts to ‘upgrade’ neonatal units with the aim that 80% of districts have an intermediate-level unit by 2025.^4^ Considerable efforts are being made to develop robust, affordable CPAP machines and other devices, to equip these units and to train staff. Our data suggest such initiatives may not achieve their intended effects on neonatal outcomes without major increases in nurse staffing in settings where nurse-to-patient ratios are low. Parallel efforts to provide sufficient human resources for health must therefore be made, which will require overcoming the severely constrained fiscal space that Kenya, like many LMICs, has for health spending.^30^ Our data also suggest some scope for supporting nurses through carefully designed, more formal task-sharing, but this will not obviate the need to substantially increase the numbers of professional nurses, as an inappropriate skill mix can also be associated with poorer health outcomes.^31^

We acknowledge that our study has some limitations. First, we obtained data using direct observation that may be subject to the Hawthorne effect, in which observed individuals modify their behaviour.^15^ We have described methods we used to minimise this effect, including a familiarisation period and observing randomly selected neonatal nursing shifts. Our data also only focused on government-owned hospitals. As nurse staffing levels are better in private and faith-based facilities our findings may not be generalisable to such settings.^14^ Even in Nairobi, the capital city, public hospitals are, however, probably responsible for as much as 70% of inpatient neonatal care, and we suggest our findings apply to settings with staff-to-patient ratios similar to those in the sites we studied.^32^

The observation tool we used measures only bedside nursing tasks. Nurses have many other responsibilities away from the bedside, including administration, supervising and teaching. Furthermore, the observers were not attempting to assess the quality of any task performed, for example they did not assess whether an intravenous drug was given safely or whether the correct amount of feed was given during a nasogastric feed. They recorded only if a task was done (and by whom) or not done at all. We are therefore limited in making inferences about the quality of bedside nursing tasks done, or the completion or quality of non-patient-facing nursing tasks.

We also employed different observers for each of the eight neonatal units in our study, and some of the differences noted may be influenced by observer bias. To minimise this risk, we developed standard operating procedures for our measurements. We trained the observers together in both classroom and bedside settings, comparing their measurements with those of the senior research team, which included nurses.

Despite these limitations, our study provides the most detailed examination of nursing care provision and workloads in low-resource neonatal care settings. We highlight the limited time available to nurses and probably unavoidably high levels of missed nursing care and nursing care delegated to unsupervised nursing students and mothers. Our findings illuminate a neglected quality of care gap in LMICs and raise important questions about the ability of improved access to technology and training alone to address persistently high neonatal mortality. Moreover, they suggest that the additional work associated with new technologies and more advanced interventions may have unintended consequences for existing nursing work, with possible implications for patient safety, unless there are substantial improvements in nurse-to-patient ratios. Increasing nurse staffing as part of efforts to scale up access to intermediate-level neonatal units is not optional; it is a necessity.

## Contributors

AI together with DG, MM, JA, and ME conceptualised the idea for this manuscript. ME, FW, DG and MM obtained the grant funding. All authors contributed to the methods for the paper. AI, VK and OO oversaw data collection with oversight provided by DG, MM, JA, FW, SF, KK & ME. All authors were involved in project administration from the start to finish of this research. AI analysed the data with support from DG, MM, JA, and ME. AI drafted the manuscript with significant contributions from all authors and under the supervision of DG, MM, JA, and ME who verified the underlying data reported in this paper. All the authors reviewed all manuscript versions and agreed on a final version for submission. AI, DG and ME assessed and verified the data.

The HIGH-Q author group comprising Peter Mwangi, Lucy Kinyua, Lydia Thuranira, Virginia Njoroge, Ngina Mwangi, Loise Mwangi, Penina Musyoka, and Zainab Kioni were responsible for study set-up, engagements with counties and hospitals for formal approvals, and project administration and they reviewed and approved this manuscript.

## Data sharing statement

The dataset that was generated for this study, including anonymised individual data that were analysed, is available upon reasonable request and a written protocol.

## Declaration of interests

ME received support for the present manuscript from an NIHR Research Grant and a Wellcome Senior Fellowship and has also received a grant from the Wellcome Discovery Award in the past 36 months. He also consulted for Save the Children USA and Oxford Policy Management and was supported by the WHO Guideline Development Group Travel to participate in a WHO-Geneva meeting to develop paediatric guidelines.

All other authors declare no competing interests.
