# Supplementary material for: Delivery of care in high mortality hospital settings: a direct observational study examining 1848 h of neonatal nursing in Kenya
Source: eClinicalMedicine. 2025 Aug 14;87:103434. doi: 10.1016/j.eclinm.2025.103434 (PMC12362405; doi:10.1016/j.eclinm.2025.103434)
Supplement: Supplementary Files S1–S5 [file mmc1.docx]

**Supplementary file 1** – Adapted structured observational checklist.

| Date of completion | | | | | \|__\|__\|/\|__\|__\|/\|__\|__\|__\|__\|  (DD/MM/YYYY) | | | | | | | | | | | |
| --- | --- | --- | --- | --- | --- | --- | --- | --- | --- | --- | --- | --- | --- | --- | --- | --- |
| Observer code | | | | | \|__\|__\| (e.g., 01) | | | | | | | | | | | |
| Subject ID | | | | | \|__\|__\|- \|__\|__\|__\| (5-digit code (first 2 digits, hospital code and last 3-digit, patient number e.g., 010 or 100) | | | | | | In-patient number | | | | | |
| Recruitment period (Tick as appropriate) | | | | | | | | Baseline (Pre-intervention)  Post-intervention_1 (nurses)  Post-intervention_2 ( ward clerks) | | | | | | | | |
| Part A – Shift information (Tick as appropriate) | | | | | | | | | | | | | | | | |
| Q 1. Shift type | | | | | | | | | | | | | | | | |
| Weekday day | | | | | | | Weekday night | | | | | | | | | |
| Weekend day | | | | | | | Weekend night | | | | | | | | | |
| Q 2. Observation period | | | | | | | | | | | | | | | | |
| Start Date  \|__\|__\|/\|__\|__\|/\|__\|__\|__\|__\|  (DD/MM/YYYY) | | | | | | | Start time  \|__\|__\| :\|__\|__\|  (Please use 24-hour timing e.g., 23:59, not 11:59pm) | | | | | | | | | |
| End Date  \|__\|__\|/\|__\|__\|/\|__\|__\|__\|__\|  (DD/MM/YYYY) | | | | | | | End time  \|__\|__\| :\|__\|__\|  (Please use 24-hour timing e.g., 23:59, not 11:59pm) | | | | | | | | | |
| Q 3. Total number of patients in the ward  (Include all babies including those in KMC and acute rooms) | | | | \|__\|__\|__\| | | | Number of babies on CPAP | | | | | | | | \|__\|__\| | |
|  |  |  |  |  |  |  | Number of babies on Oxygen | | | | | | | | \|__\|__\| | |
|  |  |  |  |  |  |  | Number of babies on IV therapy (medications/ fluids) | | | | | | | | \|__\|__\| | |
|  |  |  |  |  |  |  | Number of babies on Phototherapy | | | | | | | | \|__\|__\| | |
|  |  |  |  |  |  |  | Number of babies requiring nasogastric tube/orogastric feeding | | | | | | | | \|__\|__\| | |
|  |  |  |  |  |  |  | Number of babies requiring incubator care | | | | | | | | \|__\|__\| | |
| Q 4 Is there an extra KMC Ward/ room managed by the NBU nurses? | | | | Yes  No | | | | | Number of babies on the KMC ward  \|__\|__\| | | | | | | | |
| Q 5 Is there an acute room present in the NBU? | | | | Yes  No | | | | | How many babies are present in this room?  \|__\|__\| | | | | | | | |
| Q6. Number of nurses on current 12-hour shift | | | | Actual (Based on observations) | | | | | | | | | | | | |
|  |  |  |  | Morning shift  (07:30 – 12:30) | | |  | | Night shift  (18:30 – 07:30) | | | | | |  | |
| Comment: | | | | Day shift  (07:30 – 16:30) | | |  | | Others  __________ | | | | | |  | |
|  |  |  |  | Afternoon shift  (12:30 – 18:30) | | |  | |  | | | | | |  | |
| Q 7. Device check. On the current shift, how many of the following devices below are present and functional | | | | | | | | | | | | | | | | |
| Glucometer  (If no strips, put as 0) |  | Weighing scale | | | |  | | | | Pulse oximeter | | | | | |  |
| Thermometer  (owned by the unit) |  | Stethoscopes  **(owned by the unit)** | | | |  | | | |  | | | | | |  |
| Instruction: Please reconcile the following information below at end of the shift | | | | | | | | | | | | | | | | |
| Q 8. How many of the following processes occurred during the shift? | | | | | | | Admissions | | | | | | | \|___\| | | |
|  |  |  |  |  |  |  | Discharges | | | | | | | \|___\| | | |
|  |  |  |  |  |  |  | Referrals | | | | | | | \|___\| | | |
|  |  |  |  |  |  |  | Deaths | | | | | | | \|___\| | | |
| Q 9. How many of the following staff cadres where present on this shift? | | | | | | | Medical officer | | | | | | \|___\| | | | |
|  |  |  |  |  |  |  | Clinical Officer Interns | | | | | | \|___\| | | | |
|  |  |  |  |  |  |  | Medical officer interns | | | | | | \|___\| | | | |
|  |  |  |  |  |  |  | Nursing officer interns | | | | | | \|___\| | | | |
|  |  |  |  |  |  |  | Nursing students | | | | | | \|___\| | | | |
|  |  |  |  |  |  |  | Others  ____________ | | | | | | \|___\| | | | |
|  |  |  |  |  |  |  | Others  ____________ | | | | | | \|___\| | | | |
| Q 10. Was the ward-in-charge or deputy ward-in-charge present at any time during the 12-hour observation period | | | | | | | Yes | | | | | |  | | | |
|  |  |  |  |  |  |  | No | | | | | |  |  |  |  |
| Part B – Baby’s biodata (Tick as appropriate) | | | | | | | | | | | | | | | | |
| Q 11. Care category | | | | | | | | | | | | | | | | |
| Category A (Critical/HDU) | | | Category B (Acute) | | | | | | | | | Category C (Stable) | | | | |
| Q 12. Date of admission  \|__\|__\|/\|__\|__\|/\|__\|__\|__\|__\|  (DD/MM/YYYY) | | | | | | |  | | | | | | | | | |
| Q 13. Current age (write in days if above 24 hours old, if less than please write in hours) | | | | | | | Hours \|__\|__\| Days \|__\|__\| | | | | | | | | | |
| Q14. Gender | | | | | | |  | | | | | | | | | |
| Male Female | | | | | | |  | | | | | | | | | |
| Q 15. Current diagnosis | | | | | | | Diagnosis 1 (Primary diagnosis)  __________________________________________ | | | | | | | | | |
|  |  |  |  |  |  |  | Diagnosis 2 (Secondary diagnosis)  __________________________________________ | | | | | | | | | |
|  |  |  |  |  |  |  | Diagnosis 3 (Secondary diagnosis)  ­­­­­__________________________________________ | | | | | | | | | |
|  |  |  |  |  |  |  | Other diagnosis  ­­­­­__________________________________________ | | | | | | | | | |
| Q 16. Birth weight (grams)  \|__\|__\|__\|__\| | | | | | | | | | | | | | | | | |
| Q 17. Current weight (Most recent weight in grams)  \|__\|__\|__\|__\| | | | | | | | | | | | | | | | | |
| Q 18. Which of the following medical devices/interventions is the baby on? (Tick yes if any device applies and tick no if it does not) | | | | | | | | | | | | | | | | |
| CPAP | | | | | | | | Yes  No | | | | | | | | |
| Incubators | | | | | | | | Yes  No | | | | | | | | |
| Intravenous fluids | | | | | | | | Yes  No | | | | | | | | |
| Intravenous cannula (without fluid attached) | | | | | | | | Yes  No | | | | | | | | |
| Nasogastric/Orogastric tube | | | | | | | | Yes  No | | | | | | | | |
| Oxygen | | | | | | | | Yes  No | | | | | | | | |
| Phototherapy | | | | | | | | Yes  No | | | | | | | | |

| **Nursing tasks** | **Task frequency** | **Expected number within current 12-hour shift** | **Tasks done (Insert Y, if yes and N if no)** | **Time tasks done** | **Comment** |
| --- | --- | --- | --- | --- | --- |
| **Routine nursing care** |  |  |  |  |  |
| Nurses handing over patient |  | 1 |  |  |  |
| Patient assessment before shift |  | 1 |  |  |  |
| Nurse washes / sanitises hands before patient assessment  (Assess the first patient contact) |  | 1 |  |  |  |
| Nurse attends the ward round with doctor (s) to see patient.  (If nurse student, mark as no) |  | 1 |  |  |  |
| Nurse communicates with caregiver details of care/ counsels the caregiver |  | 1 |  |  |  |

| **Nursing tasks** | **Task frequency**  **(Circle option)** | **Expected number within current 12-hour shift** | **Tasks done**  **(Insert Y, if yes and N if no)** | **Task performed by**  **(N for nurse,**  **NS* –Student nurse, M- Caregiver/**  **Mother, W- ward clerk)** | **Time tasks done** | **Comment** |
| --- | --- | --- | --- | --- | --- | --- |
| Temperature check | 4/6/12 hourly | 1 /2 /3 |  |  |  |  |
|  |  |  |  |  |  |  |
|  |  |  |  |  |  |  |
| Pulse/heart rate check | 4/6/12 hourly | 1 /2 /3 |  |  |  |  |
|  |  |  |  |  |  |  |
|  |  |  |  |  |  |  |
| Respiratory rate | 4/6/12 hourly | 1/ 2 /3 |  |  |  |  |
|  |  |  |  |  |  |  |
|  |  |  |  |  |  |  |
| Pulse oximetry | 4/6/12 hourly | 1/ 2 /3 |  |  |  |  |
|  |  |  |  |  |  |  |
|  |  |  |  |  |  |  |

*- Task performed by a nursing student under supervision should be termed as done by a nurse

| **Nursing tasks**  (Routine newborn care) | **Task frequency**  **(Circle option)** | **Expected number within current 12-hour shift** | **Tasks done**  **(Insert Y, if yes and N if no)** | **Task performed by**  **(N for nurse,**  **NS* –Student nurse, M- Caregiver/**  **Mother, W- ward clerk)** | **Time tasks done** | **Comment** |
| --- | --- | --- | --- | --- | --- | --- |
| Cleaning baby |  | 1 |  |  |  |  |
| Linen change |  | 1 |  |  |  |  |
| Weight check |  | 1 |  |  |  |  |
| Checking incubator settings |  | 1 |  |  |  |  |
| Diaper change |  | 1 |  |  |  |  |
| Cord care |  | 1 |  |  |  |  |
| Turning the baby |  | 4 |  |  |  |  |
|  |  |  |  |  |  |  |
|  |  |  |  |  |  |  |
|  |  |  |  |  |  |  |

| **Nursing tasks**  **(Feeding)** | **Task frequency**  **(Circle option)** | **Expected number within current 12-hour shift** | **Tasks done**  **(Insert Y, if yes and N if no)** | **Task performed by**  **(N for nurse,**  **NS* –Student nurse, M- Caregiver/**  **Mother, W- ward clerk)** | **Time tasks done** | **Comment** |
| --- | --- | --- | --- | --- | --- | --- |
| Breastfeeding  Cup/spoon feeding  Nasogastric/  Oro-gastric  tube  No oral  feeding | 3-hourly | 4 |  |  |  |  |
|  |  |  |  |  |  |  |
|  |  |  |  |  |  |  |
|  |  |  |  |  |  |  |
| **NG Tube feeding process &** | | | | | | |
| Check tube positioning | 3-hourly | 4 |  |  |  |  |
|  |  |  |  |  |  |  |
|  |  |  |  |  |  |  |
|  |  |  |  |  |  |  |
| Measure feeds | 3-hourly | 4 |  |  |  |  |
|  |  |  |  |  |  |  |
|  |  |  |  |  |  |  |
|  |  |  |  |  |  |  |
| Position the baby after feeding | 3-hourly | 4 |  |  |  |  |
|  |  |  |  |  |  |  |
|  |  |  |  |  |  |  |
|  |  |  |  |  |  |  |

& - Complete for only NG-tube-fed babies

| **IV Medication** | | | | | | |
| --- | --- | --- | --- | --- | --- | --- |
| **Is the baby on IV medication? Yes (complete section below)**  **(Tick as appropriate)**  **No (cross-out section below)** | | | | | | |
| **Nursing tasks**  **(Medication)** | **Task frequency**  **(Please circle)** | **Expected number within current 12-hour shift** | **Tasks done**  **(Insert Y, if yes and N if no)** | **Time tasks done** | **Task performed by**  **(N for nurse,**  **NS* –Student nurse, M- Caregiver/**  **Mother, W- ward clerk)** | **Comment** |
| Drug name  _________________________ | 6/8/12/24  hourly | \|___\| |  |  |  |  |
|  |  |  |  |  |  |  |
| Drug name  _________________________ | 6/8/12/24  hourly | \|___\| |  |  |  |  |
|  |  |  |  |  |  |  |
| Drug name  _________________________ | 6/8/12/24  hourly | \|___\| |  |  |  |  |
|  |  |  |  |  |  |  |
| Drug name  _________________________ | 6/8/12/24  hourly | \|___\| |  |  |  |  |
|  |  |  |  |  |  |  |
| 6 hourly – QDS/QID, 8 hourly – TDS/TID, 12 hourly – BD, 24 hourly - OD | | | | | | |
| **Oral Medication** | | | | | | |
| **Is the baby on oral medication? Yes (complete section below)**  **(Tick as appropriate)**  **No (cross-out section below)** | | | | | | |
| **Nursing tasks**  **(Medication)** | **Task frequency** | **Expected number within current 12-hour shift** | **Tasks done**  **(Insert Y, if yes and N if no, E if not determined)** | **Time tasks done** | **Task performed by**  **(N for nurse,**  **NS* –Student nurse, M- Caregiver/**  **Mother, W- ward clerk )** | **Comment** |
| Drug name  _________________________ | 12/24 hourly | \|___\| |  |  |  |  |
| Drug name  _________________________ | 12/24 hourly | \|___\| |  |  |  |  |
| Drug name  _________________________ | 12/24 hourly | \|___\| |  |  |  |  |
| Drug name  _________________________ | 12/24 hourly | \|___\| |  |  |  |  |

| **IV Medication delivery process (Perform this observation for first round of medications)** | | | | |
| --- | --- | --- | --- | --- |
| **Nursing tasks**  **(Medication)** | **Tasks done**  **(Insert Y, if yes and N if no, E if not determined)** | **Not applicable e.g., if patient not on IV medication** | **Task performed by**  **(N for nurse,**  **NS* –Student nurse, M- Caregiver/**  **Mother, W- ward clerk )** | **Comments** |
| Review of treatment sheet |  |  |  |  |
| Cannula flush with saline before drug administration |  |  |  |  |
| Cannula flush with saline after drug administration |  |  |  |  |
| **Supervision of mother during Kangaroo Mother Care (KMC)** | | | | |
| **Is the baby on KMC? Yes (complete section below)**  **(Tick as appropriate)**  **No (cross-out section below)** | | | | |
| Supervision of mother during KMC |  |  |  |  |

| **Phototherapy** | | | | | | |
| --- | --- | --- | --- | --- | --- | --- |
| **Is the baby on oral medication? Yes (complete section below)**  **(Tick as appropriate)**  **No (cross-out section below)** | | | | | | |
| **Nursing tasks**  **(Special newborn care)** | **Task frequency** | **Expected number within current 12-hour shift** | **Tasks done**  **(Y-yes, N- no)** |  | **Task performed by**  **(N for nurse,**  **NS* –Student nurse, M- Caregiver/**  **Mother, W- ward clerk )** | **Comment** |
| Turning/positioning | 4 hourly | 3 |  |  |  |  |
|  |  |  |  |  |  |  |
|  |  |  |  |  |  |  |
| Skin assessment | 6 hourly | 2 |  |  |  |  |
|  |  |  |  |  |  |  |
| Eye care | 12 hourly | 1 |  |  |  |  |
| Changing eye pads | 12 hourly | 1 |  |  |  |  |

| **Continuous Positive Airway Pressure** | | | | |  |
| --- | --- | --- | --- | --- | --- |
| **Is the baby on Continuous Positive Airway Pressure? Yes (complete section below)**  **(Tick as appropriate)**  **No (cross-out section below)** | | | | | |
| **Nursing tasks**  **(Medication)** | **Task frequency** | **Expected number within current 12-hour shift** | **Tasks done**  **(Insert Y, if yes and N if no, E if not determined)** | **Task performed by**  **(N for nurse,**  **NS* –Student nurse, M- Caregiver/**  **Mother, W- ward clerk )** | **Comment** |
| Checking nasal prong position | 4 hourly | 3 |  |  |  |
|  |  |  |  |  |  |
|  |  |  |  |  |  |
| Checking oxygen flow rate | 4 hourly | 3 |  |  |  |
|  |  |  |  |  |  |
|  |  |  |  |  |  |

| **Oxygen therapy** | | | | |  |
| --- | --- | --- | --- | --- | --- |
| **Is the baby on oxygen therapy? Yes (complete section below)**  **(Tick as appropriate)**  **No (cross-out section below)** | | | | | |
| **Nursing tasks**  **(Medication)** | **Task frequency** | **Expected number within current 12-hour shift** | **Tasks done**  **(Insert Y, if yes and N if no, E if not determined)** | **Task performed by**  **(N for nurse,**  **NS* –Student nurse, M- Caregiver/**  **Mother, W- ward clerk )** | **Comment** |
| Checking nasal prong position | 4 hourly | 3 |  |  |  |
|  |  |  |  |  |  |
|  |  |  |  |  |  |
| Checking oxygen flow rate | 4 hourly | 3 |  |  |  |
|  |  |  |  |  |  |
|  |  |  |  |  |  |

| **Documentation** | | | | | |
| --- | --- | --- | --- | --- | --- |
| **Documentation of tasks (source)** | **Done**  **(Please tick)** | **Not Done**  **(Please tick)** | **Not applicable**  **(Please tick)** | **If done, how many times**  **(Please write number)** | **Comments** |
| Neonatal assessment by nurse (Nursing cardex) |  |  |  |  |  |
| Nursing care plan (Nursing cardex/ care plan) |  |  |  |  |  |
| Temperature  measurements |  |  |  |  |  |
| Heart rate |  |  |  |  |  |
| Respiratory rate |  |  |  |  |  |
| Oxygen saturation |  |  |  |  |  |
| Ward round details |  |  |  |  |  |
| Frequency and volume of feed |  |  |  |  |  |
| Health talks/ Communication to parents |  |  |  |  |  |
| Volume of IV fluids |  |  |  |  |  |
| Weight check |  |  |  |  |  |

| 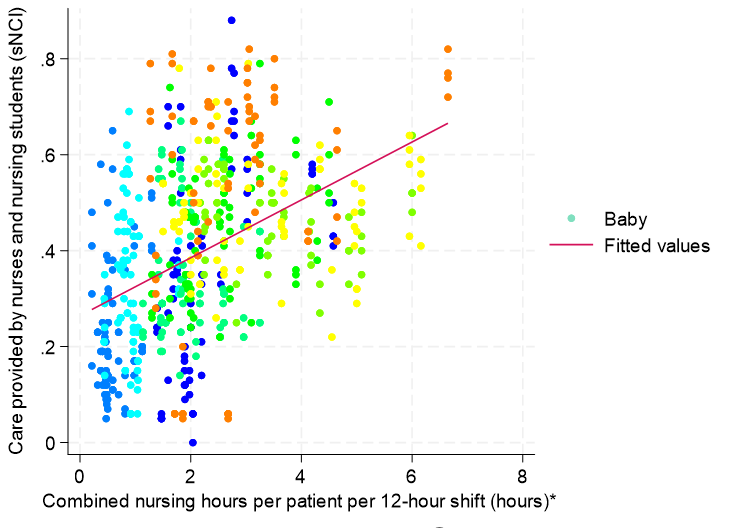  Supplemental File 2 – Scatter plot showing the relationship between combined (nurses and nursing students) nursing hours per patient per 12-hour shift and proportion of care provided by both nurses and nursing students (sNCI) (Spearman’s correlation =0.48)  *- Combined nursing hours per patient per 12-hour shift includes nursing student hours in the measure of nursing hours per patient per 12-hr shift  The individual colours represent each of the 8 neonatal units  Supplemental File 3 – Specific nursing tasks disaggregated by patient care category and whether the task was performed by the nurse or others or missed. | | | | | | | | | | | | | |
| --- | --- | --- | --- | --- | --- | --- | --- | --- | --- | --- | --- | --- | --- |
| **Domains** | **Task** | **Category A babies** | | | | **Category B babies** | | | | **Category C babies** | | | |
|  |  | n | Nurse | Others (Nursing student/mother) | Missed | n | Nurse | Others (Nursing student/mother) | Missed | n | Nurse | Others (Nursing student/mother) | Missed |
| Routine nursing care | Patient handover | 202 | **99.5** [94.8, 100.0] | 0.0 [0.0, 0.0] | 0.5 [0.0, 5.2] | 187 | **95.2** [63.9, 99.6] | 0.0 [0.0, 0.0] | 4.8 [0.4, 36.1] | 208 | **90.4** [66.2, 97.8] | 0.0 [0.0, 0.0] | 9.6 [2.2, 33.8] |
|  | Patient assessment before a shift | 202 | **90.1** [57.3, 98.4] | 0.0 [0.0, 0.0] | 9.9 [1.6, 42.7] | 186 | **83.3** [44.8, 96.9] | 0.0 [0.0, 0.0] | 16.7 [3.1, 55.2] | 208 | **70.2** [42.0, 88.5] | 0.0 [0.0, 0.0] | 29.8 [11.5, 58.0] |
|  | Hand washing | 197 | **61.9** [32.4, 84.7] | 0.0 [0.0, 0.0] | 38.1 [15.3, 67.6] | 180 | **56.7** [22.3, 85.6] | 0.0 [0.0, 0.0] | 43.3[14.4, 77.7] | 190 | **45.3** [15.6, 78.7] | 0.0 [0.0, 0.0] | 54.7 [21.3, 84.4] |
|  | Ward round attendance | 68 | **22.1** [10.2, 41.4] | 0.0 [0.0, 0.0] | 77.9 [58.6, 89.8] | 44 | **11.4** [1.3, 56.2] | 0.0 [0.0, 0.0] | 88.6 [43.8, 98.7] | 44 | **6.8** [1.1, 33.1] | 0.0 [0.0, 0.0] | 93.2 [66.9, 98.9] |
|  | Patient communication | 200 | **55.5** [33.7, 75.4] | 0.0 [0.0, 0.0] | 44.5 [24.6, 66.3] | 186 | **46.2** [20.1, 74.6] | 0.0 [0.0, 0.0] | 53.8 [25.4, 79.9] | 204 | **38.7** [21.4, 59.5] | 0.0 [0.0, 0.0] | 61.3 [40.5, 78.6] |
| Vital sign monitoring^a^ | Temperature | 200 | **22.5** [7.8, 50.0] | 50.5 [26.9, 73.9] | 27.0 [13.0, 47.9] | 186 | **3.2** [0.9, 11.3] | 60.8 [28.1, 86.0] | 36.0 [12.2, 69.5] | 208 | **2.4** [0.3, 14.9] | 52.9 [26.7, 77.6] | 44.7 [19.9, 72.4] |
|  | Respiratory rate | 200 | **26.5** [11.2, 50.9] | 48.0 [23.5, 73.5] | 25.5 [15.6, 38.8] | 186 | **3.2** [1.0, 10.0] | 50.5 [22.3, 78.4] | 46.2 [19.9, 74.9] | 208 | **3.4** [0.7, 15.4] | 30.8 [13.5, 55.8] | 65.9 [42.2, 83.6] |
|  | Pulse rate | 201 | **28.9** [12.6, 53.3] | 55.2 [28.1, 79.5] | 15.9 [6.7, 33.2] | 186 | **2.7** [0.6, 10.7] | 61.3 [26.0, 87.7] | 36.0 [10.2, 73.7] | 208 | **4.3**[1.3, 13.2] | 46.6 [18.7, 76.8] | 49.0 [19.7, 79.1] |
|  | Pulse oximetry | 177 | **33.3** [15.3, 58.1] | 48.0 [23.1, 74.0] | 18.6 [8.3, 36.7] | 171 | **4.1** [1.4, 11.3] | 57.9 [23.1, 86.3] | 38.0 [11.0, 75.3] | 184 | **4.3** [1.1, 16.2] | 41.8  [13.7, 76.6] | 53.8 [20.3, 84.2] |
| Supplemental File 3 (continued) – Specific nursing tasks disaggregated by patient care category and whether the task was performed by the nurse, or others or missed (n=597). | | | | | | | | | | | | | |
| **Domains** | **Task** | **Category A babies** | | | | **Category B babies** | | | | **Category C babies** | | | |
|  |  | n | Nurse | Others (Nursing student/mother) | Missed | n | Nurse | Others (Nursing student/mother) | Missed | n | Nurse | Others (Nursing student/mother) | Missed |
| Routine newborn care^b^ | Cleaning baby | 152 | **0.7** [0.1, 6.7] | 67.1 [41.1, 85.6] | 32.2 [13.6, 59.0] | 132 | **0.0** [0.0, 0.0] | 60.6 [24.9, 87.7] | 39.4 [12.3, 75.1] | 137 | **0.7** [0.1, 7.2] | 59.1 [23.8, 87.0] | 40.1 [12.6, 75.8] |
|  | Linen change | 172 | **2.9** [1.1, 7.2] | 47.1 [27.5, 67.6] | 50.0 [28.8, 71.2] | 154 | **0.0** [0.0, 0.0] | 33.8 [14.1, 61.2] | 66.2 [38.8, 85.9] | 153 | **2.6** [0.7, 9.4] | 35.3 [13.1, 66.5] | 62.1 [32.1, 85.0] |
|  | Weight check**^c^** | 79 | **2.5** [0.6, 9.8] | 79.7 [69.3, 87.3] | 17.7 [10.7, 27.9] | 57 | **0.0** [0.0, 0.0] | 80.7 [47.8, 95.0] | 19.3 [5.0, 52.2] | 53 | **0.0** [0.0, 0.0] | 90.6 [78.9, 96.1] | 9.4 [3.9, 21.1] |
|  | Checking incubator settings | 77 | 22.1 [14.1, 32.9] | 50.6 [39.5, 61.8] | 27.3 [18.4, 38.4] | 4 | 75.0 [7.1, 99.2] | 25.0 [0.8, 92.9] | 0.0 [0.0, 0.0] | 0 | 0.0 [0.0, 0.0] | 0.0 [0.0, 0.0] | 0.0 [0.0, 0.0] |
|  | Diaper change | 202 | **2.0** [0.8, 4.8] | 95.5 [86.4, 98.6] | 2.5 [0.5, 11.5] | 187 | **0.0** [0.0, 0.0] | 98.4 [91.9, 99.7] | 1.6 [0.3, 8.1] | 208 | **1.4** [0.5, 4.4] | 95.7 [82.8, 99.0] | 2.9 [0.4, 17.5] |
|  | Cord care | 138 | **0.7**  [0.1, 8.7] | 53.6  [29.3, 76.4] | 45.7  [22.8, 70.6] | 115 | **1.7**  [0.4, 8.0] | 53.9  [26.2, 79.4] | 44.3 [18.9, 73.2] | 91 | **0.0** [0.0, 0.0] | 64.8  [24.4, 91.3] | 35.2  [8.7, 75.6] |
| Physical turning^a^ | Physical turning | 200 | **3.5**  [1.1, 10.3] | 90.5 [75.0, 96.8] | 6.0  [1.5, 20.9] | 186 | **1.6** [0.3, 8.2] | 97.3 [91.3, 99.2] | 1.1 [0.2, 4.7] | 207 | **1.4** [0.5, 4.4] | 97.6 [90.8, 99.4] | 1.0 [0.1, 9.3] |
| Cup feeding^a^ | Cup feeding | 15 | **6.7** [0.8, 39.7] | 93.3 [60.3, 99.2] | 0.0 [0.0, 0.0] | 64 | **1.6** [0.2, 10.6] | 96.9 [88.1, 99.3] | 1.6 [0.2, 10.6] | 98 | **4.1** [1.5, 10.5] | 93.9 [86.9, 97.3] | 2.0 [0.5, 7.9] |
| Nasogastric tube feeding | Nasogastric tube feeding | 141 | 0.0 [0.0, 0.0] | 95.0 [89.9, 97.6] | 5.0 [2.4, 10.1] | 53 | 0.0 [0.0, 0.0] | 98.1 [87.3, 99.7] | 1.9 [0.3, 12.7) | 0 | 0.0 [0.0, 0.0] | 0.0 [0.0, 0.0] | 0.0 [0.0, 0.0] |
|  | Checking tube position pre-feed | 143 | 4.2 [1.9, 9.1] | 45.5 [37.4, 53.7] | 50.3 [42.1, 58.5] | 53 | 0.0 [0.0, 0.0] | 52.8 [39.2, 69.0] | 47.2 [34.0, 60.8] | 0 | 0.0 [0.0, 0.0] | 0.0 [0.0, 0.0] | 0.0 [0.0, 0.0] |
|  | Measure feeds | 142 | 0.0 [0.0, 0.0] | 95.1 [90.0, 97.6] | 4.9 [2.4, 10.0] | 53 | 0.0 [0.0, 0.0] | 98.2 [87.7, 99.8] | 1.8 [0.2, 12.2] | 0 | 0.0 [0.0, 0.0] | 0.0 [0.0, 0.0] | 0.0 [0.0, 0.0] |
|  | Positioning babies after the feed | 142 | 0.7 [0.1,4.9] | 87.3 [80.7, 91.9] | 12.0 [7.5, 18.5] | 53 | 0.0 [0.0, 0.0] | 96.2 [85.7, 99.1] | 3.8 [0.1, 14.3] | 0 | 0.0 [0.0, 0.0] | 0.0 [0.0, 0.0] | 0.0 [0.0, 0.0] |
| Medication | Oral medication^a^ | 31 | 9.7 [3.0, 27.0] | 61.3 [42.7, 77.1] | 29.0 [15.4, 47.9] | 12 | **16.7** [3.5, 52.4] | 75.0 [40.9, 92.9] | 8.3 [0.9, 47.5] | 31 | **6.5** [0.2, 23.5] | 67.7 [48.9, 82.2] | 25.8 [13.0, 44.6] |
|  | Intravenous medication | 67 | 40.3 [29.1, 52.6] | 50.7 [38.7, 62.7] | 9.0 [4.0, 18.8] | 74 | 54.1 [42.5, 65.2) | 36.5 [26.2, 48.2] | 9.5 [4.5, 18.7] | 0 | 0.0 [0.0, 0.0] | 0.0 [0.0, 0.0] | 0.0 [0.0, 0.0] |
| Phototherapy | Turning/positioning | 14 | 7.1 [0.8, 42.0] | 92.9 [58.0, 99.2] | 0.0 [0.0, 0.0] | 25 | 12.0 [3.7, 32.7] | 84.0 [63.0, 94.2] | 3.9 [0.5, 25.5] | 2 | 0.0 [0.0, 0.0] | 50.0 [-] | 50.0 [-] |
|  | Skin assessments | 14 | 42.9 [18.9, 70.7] | 21.4 [6.3, 52.7] | 35.7 [14.3, 65.0] | 24 | 29.2 [14.0, 51.0] | 16.7 [6.1, 38.3] | 54.2 [33.6, 73.4] | 2 | 50.0 [-] | 50.0 [-] | 0.0 [0.0, 0.0] |
|  | Eye care | 14 | 28.6 [10.0, 58.9] | 57.1 [29.3, 81.1] | 14.3 [3.1, 46.5] | 23 | 34.8 [17.7, 56.9) | 30.4 [14.6, 52.8] | 34.8 [17.7, 56.9] | 1 | 0.0 [0.0, 0.0] | 100.0  [-] | 0.0 [0.0, 0.0] |
|  | Changing eye pad | 14 | 7.1 [0.8, 42.0] | 14.3 [3.1, 46.5] | 78.6 [47.3, 93.7] | 22 | 36.4 [18.5, 59.0] | 0.0 [0.0, 0.0] | 63.6 [41.0, 81.5] | 2 | 50.0 [-] | 50.0 [-] | 0.0 [0.0, 0.0] |
| Continuous Positive Airway Pressure | Checking nasal prong position | 13 | 38.5 [15.3, 68.4] | 46.2 [20.3, 74.2) | 15.4 [3.3, 49.3] | 0 | 0.0 [0.0, 0.0] | 0.0 [0.0, 0.0] | 0.0 [0.0, 0.0] | 0 | 0.0 [0.0, 0.0] | 0.0 [0.0, 0.0] | 0.0 [0.0, 0.0] |
|  | Checking oxygen flow rate | 13 | 53.8 [25.8, 79.7) | 15.4 [3.3, 49.3] | 30.8 [10.7, 62.2] | 0 | 0.0 [0.0, 0.0] | 0.0 [0.0, 0.0] | 0.0 [0.0, 0.0] | 0 | 0.0 [0.0, 0.0] | 0.0 [0.0, 0.0] | 0.0 [0.0, 0.0] |
| Oxygen therapy | Checking nasal prong position | 64 | 20.3 [12.0, 32.2] | 78.1 [66.1, 86.7] | 1.6 [0.2, 10.6] | 0 | 0.0 [0.0, 0.0] | 0.0 [0.0, 0.0] | 0.0 [0.0, 0.0] | 0 | 0.0 [0.0, 0.0] | 0.0 [0.0, 0.0] | 0.0 [0.0, 0.0] |
|  | Checking oxygen flow rate | 65 | 21.5 [13.1, 33.4] | 55.4 [43.0, 67.1] | 23.1 [14.3, 35.1] | 0 | 0.0 [0.0, 0.0] | 0.0 [0.0, 0.0] | 0.0 [0.0, 0.0] | 0 | 0.0 [0.0, 0.0] | 0.0 [0.0, 0.0] | 0.0 [0.0, 0.0] |

NA – Not applicable, a -Other group is majorly mothers, b - Other group is majorly nursing students c- Other group is majorly nutritionists.

Emboldened numbers in the table show the percentage of the particular tasks performed by nurses and how these change across patient care categories.

Supplemental File 4 – Model diagnostics to evaluate for heteroskedasticity. Plot of combined nursing hours and standardized residuals.

Supplemental File 5 – Model diagnostics to evaluate for normality. Q-Q plot to assess normality of level 1 residuals
